# Pessimism is associated with greater all-cause and cardiovascular mortality, but optimism is not protective

**DOI:** 10.1038/s41598-020-69388-y

**Published:** 2020-07-28

**Authors:** John B. Whitfield, Gu Zhu, J. George Landers, Nicholas G. Martin

**Affiliations:** 1 Genetic Epidemiology, QIMR Berghofer Medical Research Institute, 300 Herston Road, Brisbane, QLD 4006 Australia; 2 Chania, Crete Greece

**Keywords:** Psychology, Medical research, Risk factors

## Abstract

Scores on an optimistic-pessimistic personality scale have been associated with mortality, but optimism and pessimism scores are separable traits and it is unclear which has effects on health or longevity. The Life Orientation Test (LOT), containing items for optimism and pessimism, was included in a twin study on health of Australians aged over 50 in 1993–1995. After a mean of 20 years, participants were matched against death information from the Australian National Death Index. 1,068 out of 2,978 participants with useable LOT scores had died. Survival analysis tested for associations between separate optimism and pessimism scores and mortality from any cause, and from cancers, cardiovascular diseases or other known causes. Age-adjusted **s**cores on the pessimism scale were associated with all-cause and cardiovascular mortality (Hazard Ratios per 1 standard deviation unit, 95% confidence intervals and p-values 1.134, 1.065–1.207, 8.85 × 10^–5^ and 1.196, 1.045–1.368, 0.0093, respectively) but not with cancer deaths. Optimism scores, which were only weakly correlated with pessimism scores (age-adjusted rank correlation = − 0.176), did not show significant associations with overall or cause-specific mortality. Reverse causation (disease causing pessimism) is unlikely because in that case both cardiovascular diseases and cancers would be expected to lead to pessimism.

## Introduction

Mortality in economically advanced countries has been in long-term decline but concern has been expressed about pauses or reversals in this trend. Adverse changes may be related to poor economic circumstances for the whole population or sub-groups, leading to loss of hope^1,2^. At the individual level, studies have shown associations between the personality characteristic of optimism/pessimism and risk of disease. There are reported associations with coronary heart disease^3,4^, stroke^5^, all-cause mortality^6,7^, or all-cause mortality with sub-division into grouped causes of death^8–10^. A recent meta-analysis^11^ confirmed these associations. In general, more optimistic people have longer survival than more pessimistic people.

Many of these epidemiological studies measured the traits of optimism and pessimism using the Life Orientation Test (LOT^12^). This measures outcome expectancies by recording agreement or disagreement with optimistic and pessimistic statements. The originators of this instrument^13^ view optimism/pessimism as an important and stable dimension of personality correlated with but distinguishable from hopelessness, depression, self-esteem and locus of control. Importantly from our perspective, they also comment^13^ that “… optimism and pessimism may not anchor the two ends of one dimension. Rather, they may reflect two related, but separate, dimensions.”. However “… the vast majority of existing research has treated optimism and pessimism as bipolar in nature”.

A previous publication from our group^14^ found an association between scores on the combined optimism/pessimism scale and all-cause mortality. This was based on data from the subjects described here, but with death information only up to the end of 2009. Our current analysis focuses on differentiating optimism and pessimism through separate scores for each; classifies causes of death into cancers, cardiovascular diseases, and other causes; and has additional power because of the longer follow-up.

## Results

**Figure 1:**
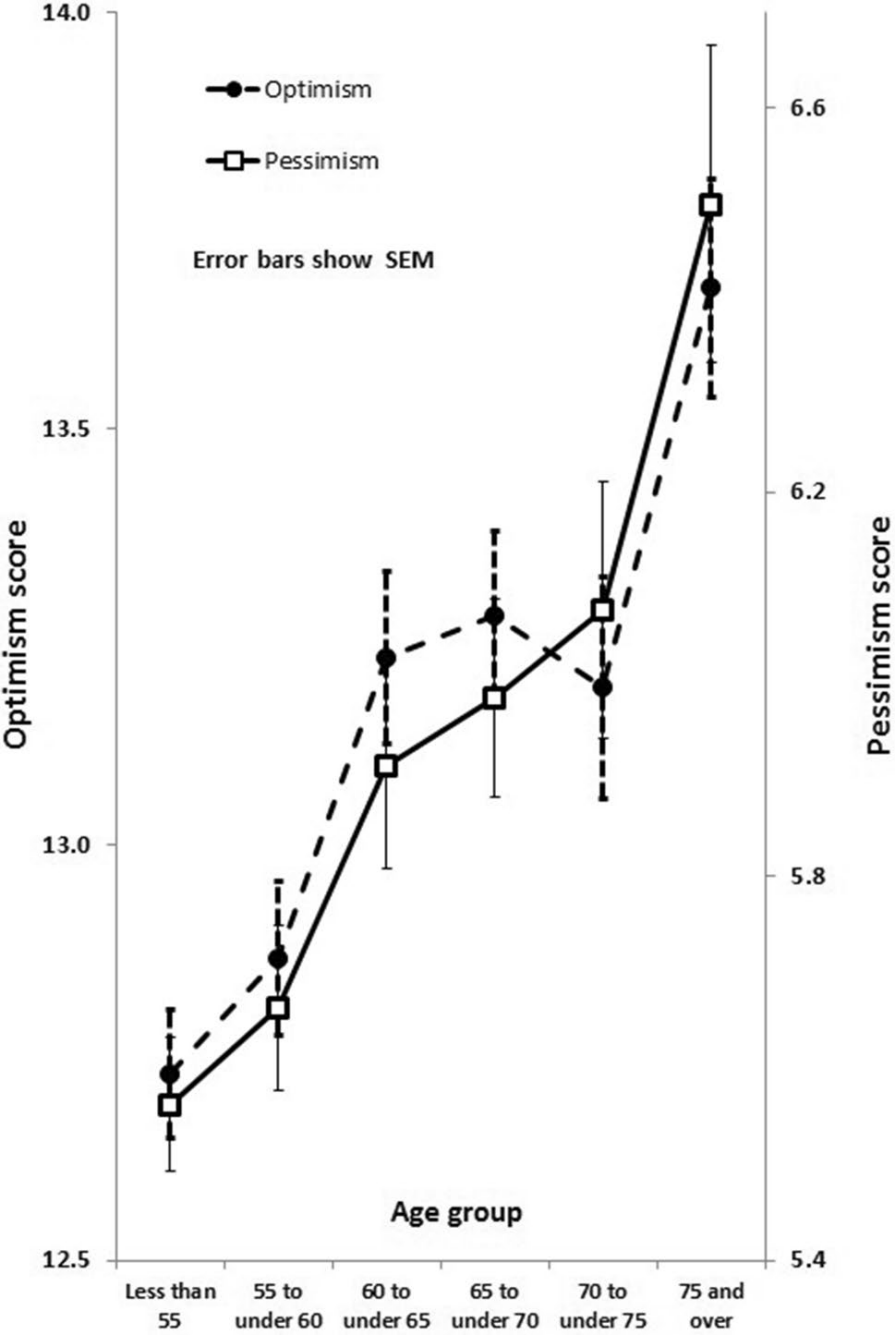
Means for optimism and pessimism scores, by age.

There were no significant differences in optimism or pessimism scores between men and women, but both increased with increasing age, as shown in Fig. 1. Distributions of sex- and age-adjusted standardised residuals of scores for optimism and pessimism are shown in Supplementary Fig. 1. Correlations between scores were statistically significant but weak: rank correlation of age- and sex-adjusted optimism and pessimism scores was − 0.176, p = 3.95 × 10^–20^; correlations with depression score were − 0.193, p = 3.27 × 10^–24^ for optimism and 0.145, p = 7.7 × 10^–14^ for pessimism. Heritabilities of optimism and pessimism scores were each around 30% (see Supplementary Table 1).

Mean and median follow-up times, to either death or censoring, were 20.0 and 23.4 years (range 0.1–23.4 years). 36% of participants with optimism or pessimism scores died during follow-up, with 99% of deaths occurring more than 1 year after completion of the questionnaire and 94% more than 3 years.

Results of survival analysis, using standardised residuals of the scores after adjusting for age and sex, are shown in Table 1. Pessimism score was significantly associated with all-cause mortality, with a Hazard Ratio of 1.134 per 1 standard deviation (SD) unit increase (95% CI 1.065–1.207).

**Table 1:** Survival analysis (Cox regression, using STATA with familial clustering) for optimism, pessimism, combined optimism/pessimism and depression scores with sex, and with sex, BMI, alcohol intake and smoking status as additional predictors.

	With sex					With sex, BMI, alcohol, smoking				
	N total	N deaths	HR	95% CI	*p *value	N total	N deaths	HR	95% CI	*p* value
**All-cause**										
Optimism score	2,819	1,036	0.975	0.913–1.042	0.458	2,444	875	0.995	0.925–1.069	0.882
Pessimism score	2,742	994	1.134	1.065–1.207	8.85 × 10^–5^	2,375	840	1.160	1.083–1.242	2.04 × 10^–5^
Combined score	2,689	969	0.912	0.855–0.973	0.0056	2,340	824	0.911	0.848–0.979	0.011
Depression score	2,754	993	1.085	1.015–1.160	0.017	2,393	842	1.081	1.006–1.162	0.034
Optimism score	2,590	912	1.013	0.941–1.089	0.738	2,265	781	1.050	0.969–1.138	0.235
Pessimism score			1.113	1.038–1.193	0.0025			1.151	1.068–1.240	2.33 × 10^–4^
Depression score			1.054	0.980–1.134	0.157			1.057	0.977–1.142	0.166
**Any cancer**										
Optimism score	2,819	313	1.063	0.937–1.205	0.342	2,444	270	1.069	0.943–1.213	0.296
Pessimism score	2,742	303	1.031	0.921–1.153	0.598	2,375	260	1.018	0.896–1.158	0.783
Combined score	2,689	297	1.018	0.903–1.148	0.766	2,340	254	1.032	0.910–1.171	0.626
Depression score	2,754	302	1.036	0.914–1.175	0.579	2,393	265	1.003	0.865–1.163	0.964
Optimism score	2,590	285	1.067	0.930–1.224	0.357	2,265	248	1.074	0.934–1.235	0.318
Pessimism score			1.046	0.923–1.186	0.479			1.045	0.907–1.203	0.544
Depression score			1.038	0.905–1.191	0.596			1.016	0.867–1.191	0.845
**Cardiovascular**										
Optimism score	2,819	237	0.928	0.811–1.061	0.274	2,444	195	0.946	0.816–1.097	0.463
Pessimism score	2,742	226	1.196	1.045–1.368	0.0093	2,375	189	1.247	1.075–1.447	0.0035
Combined score	2,689	222	0.858	0.752–0.980	0.024	2,340	187	0.855	0.737–0.991	0.038
Depression score	2,754	220	1.146	0.991–1.326	0.067	2,393	183	1.148	0.990–1.330	0.067
Optimism score	2,590	204	1.006	0.867–1.167	0.939	2,265	173	1.055	0.897–1.240	0.519
Pessimism score			1.164	1.001–1.354	0.049			1.223	1.038–1.442	0.016
Depression score			1.115	0.964–1.291	0.144			1.116	0.965–1.291	0.140
**Other causes**										
Optimism score	2,819	357	0.984	0.881–1.100	0.781	2,444	298	0.996	0.881–1.125	0.943
Pessimism score	2,742	337	1.138	1.018–1.272	0.022	2,375	281	1.180	1.041–1.337	0.0096
Combined score	2,689	325	0.918	0.821–1.026	0.133	2,340	275	0.904	0.795–1.026	0.118
Depression score	2,754	343	1.113	0.996–1.244	0.060	2,393	283	1.137	1.003–1.290	0.045
Optimism score	2,590	301	1.026	0.901–1.168	0.696	2,265	255	1.059	0.920–1.218	0.424
Pessimism score			1.079	0.950–1.224	0.242			1.118	0.972–1.286	0.119
Depression score			1.086	0.956–1.234	0.203			1.114	0.970–1.279	0.126

Firstly optimism, pessimism and depression scores individually, and then with all three together.

HR, hazard ratio per 1 SD change in score; 95% CI, 95% confidence intervals for HR.

Results in Table 1 were based on all available information, comprising five items for optimism and four for pessimism. Results were similar when scores from the original LOT (four items for optimism and four for pessimism) or the revised LOT-R (three items for each) were used. We also tested whether using time from baseline to date of death or censoring as the time dimension instead of age at death or censoring, with age at baseline as a covariate, gave similar results. Again the outcome was that pessimism score showed a significant association with mortality but optimism score did not. Results from these sensitivity analyses are shown in Supplementary Table 2.

**Figure 2:**
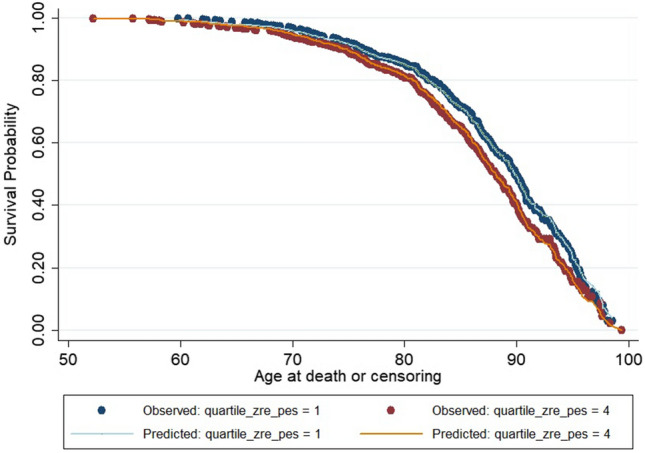
Kaplan–Meier survival curves for all-cause mortality (open and filled circles), overlaid with Cox regression estimates (continuous lines) comparing lowest (Q1, blue circles and line) and highest (Q4, red circles and orange line) quartiles of age- and sex-adjusted pessimism score (quartile_zre_pes). Median survival was 90.1 years for Q1 and 88.3 years for Q4.

Survival curves for the lowest and highest quartiles of the age- and sex-adjusted pessimism scores are shown in Fig. 2. Comparing the fourth (most pessimistic) quartile against the first (least pessimistic) quartile gave a Hazard Ratio of 1.30 and a difference in median survival of 1.8 years (88.3 versus 90.1).

The pessimism score also showed significant association with cardiovascular mortality, and with mortality from other (non-cancer) causes, but not with cancer mortality. Optimism scores, on the other hand, did not show significant associations with all-cause mortality or with the cause-of-death sub-groups. Adding body mass index, alcohol consumption and smoking status as potential predictors of survival (in addition to sex) made little difference to the estimated Hazard Ratios (see Table 1).

Because of the potential overlap between pessimism and depression, we re-analysed the survival data using depression, optimism and pessimism scores in a multivariate analysis. These results are also shown in Table 1. Depression score on its own was significantly associated with all-cause and cardiovascular mortality but when all three scores were included depression score became non-significant and the Hazard Ratio for pessimism score was still significantly above 1.00 for both all-cause and cardiovascular mortality.

## Discussion

Separation of optimism and pessimism, by calculating the two scores rather than a combination, led to several insights. Most importantly, higher pessimism score was associated with increased mortality but there was no significant association between optimism score and mortality.

The correlation between optimism and pessimism scores in our subjects is weak and they have different effects on survival. As discussed in^13^ they are not direct opposites. In addition, they change in the same direction with age rather than one going up as the other goes down. There is known to be variation in the correlation between optimism and pessimism scores with age, with negative correlations in young people diminishing gradually between the ages of 20 and 60, and no or slightly positive correlations in those over 60^15^. There is previous evidence^16,17^ for different (genetic or environmental) sources of variation, and although each showed significant heritability in our data (see Supplementary Table 1) the genetic correlation between them was weak. We can only speculate as to why both optimism and pessimism scores are higher in the older participants in this study, and note that we only have cross-sectional rather than longitudinal data on variation with age. It is possible that older people have come to a more definite conclusion about their expectations from life, whether for good or ill, and this is reflected in higher scores.

Several previous studies have found associations between the personality characteristics of optimism and pessimism, or a combination of the two, and all-cause or cause-specific mortality^3–6,8–10^. Our results suggest that combining optimism and pessimism into a single score and testing this against an outcome is inappropriate, at least when using the LOT questions. This is reinforced by results of the Finnish study of coronary heart disease^4^, which found significant associations for pessimism but not for optimism, and by the French study on stroke^5^ which found a halving of risk in the least pessimistic quartile of participants compared to the highest quartile. Others, including a large study based on the Nurses’ Health Study cohort^10^ which found significant associations with multiple conditions including both cancers and heart disease, did not use separate scales for optimism and pessimism.

Our current pessimism Hazard Ratio estimate for all-cause mortality differs from that reported earlier^14^ on the same study participants. The two estimates are not numerically comparable, because to quote from the earlier paper “Optimism/pessimism was scored as a one-factor bipolar scale, with a high score indicating pessimism”. Moreover the units for our HR estimates are z-scores (per 1 SD unit) from standardised residuals after age and sex adjustment, again not directly comparable to the earlier report.

If personality characteristics such as optimism and pessimism are to affect long-term health and survival, they must be reasonably constant over a substantial period of time. This does appear to be the case for scores based on the LOT because at least the combined optimism/pessimism score is reported to have good test–retest reliability. Intra-class correlation was 0.79 in college students, 0.75 in alcohol treatment patients, 0.72 in opiate addicts in treatment, each over comparatively short timespans, but also high at 0.71 over 10 years in middle-aged women (reviewed in^13^). It was 0.64 over 4 years in the Nurses’ Health Study cohort, for participants with a mean age of 70 at the time of the initial LOT assessment^10^. These independent repeatability estimates are notably higher than our estimates of heritability, suggesting that factors which are individual-specific (not shared by co-twins) but consistent over time play a part in determining optimistic and pessimistic attitudes.

Because our data only included a single set of responses for each person, the operation of regression dilution means that the true effect of pessimism on mortality is likely to be greater than our estimate of approximately 15% increase in risk per 1 SD increase in age-adjusted pessimism score. We consider that pessimism affects mortality, rather than ill-health leading to pessimism, because the association was found for cardiovascular deaths and deaths from ‘other causes’ but not from cancers, and because of the long period of follow-up.

Our study has both strengths and limitations. The data were obtained on only one occasion for each participant, and depend on the assumption that the scores represent characteristics with long-term stability. This assumption is justified by data from other sources, and by the significant mortality associations which we found. The data were gathered in Australia, from people who had volunteered for the baseline study during the 1990s, and associations could differ across place or time. The associations between optimism, pessimism and mortality may be different in younger people, in whom the most frequent causes of mortality will differ from those in our subjects. There are larger studies, including from other countries, and some which have included a wider range of psychological scores or known biological risk factors. We have focused on optimism and pessimism scores as predictors, with some testing of whether depression, or obesity, alcohol use and smoking, can account for our results.

Finally, and looking towards implications for health improvement, the association between pessimistic attitudes and increased mortality raises the question of whether there could be practical benefits in training people out of pessimism, and of course whether that can be achieved. Trials of potential interventions have tended to focus on patients with diagnosed disease rather than pessimistic individuals from the general population, and they have measured changes in reported attitude rather than long-term outcomes. A meta-analysis^18^ concluded that interventions can increase optimism, but although that may be desirable in itself our results suggest that improved objective outcomes may depend more on whether there are ways to reduce pessimism.

## Subjects and methods

### The older Australians study

A questionnaire-based study on health in older people was undertaken between 1993 and 1995, as described in^14,19^. Participants gave informed consent to the data collection and storage. This project was approved in accordance with the National Health and Medical Research Council’s *National Statement on Ethical Conduct in Human Research* (https://www.nhmrc.gov.au/about-us/publications/national-statement-ethical-conduct-human-research-2007-updated-2018) by both the QIMR Berghofer Medical Research Institute Human Research Ethics Committee and (for the National Death Index search) the Australian Institute of Health and Welfare (AIHW) Ethics Committee.

2,281 pairs of twins aged over 50 and living in Australia were invited to participate. The questionnaire included a range of psychological scales, and lifestyle measures assessing smoking, alcohol consumption and physical activity. 71% of those approached (1279 complete twin pairs, and 558 people whose co-twin did not participate) responded. Respondents comprised 2,197 females (response rate 75%) and 919 males (63%). The mean age of respondents at baseline was 61.5 ± 8.7 years, range 50–94 years.

### Optimism and pessimism scales

In its original published form, the LOT consisted of 8 items. A ninth item ("Overall, I expect more good things to happen to me than bad") was added for experimental purposes (Scheier, personal communication). This item was included in our survey and has been included in the computation of our optimism score.

The relevant items, which were distributed among other questions, are:

‘Optimistic’ statements

- I'm a believer in the idea that "every cloud has a silver lining"
- I'm always optimistic about my future (R)
- I always look on the bright side of things
- In uncertain times, I usually expect the best (R)
- Overall, I expect more good things to happen to me than bad (R)

‘Pessimistic’ statements

- I rarely count on good things happening to me (R)
- I hardly ever expect things to go my way (R)
- If something can go wrong for me, it will (R)
- Things never work out the way I want them to.

These were scored as No = 1, Don't Know = 2, Yes = 3.

All nine items were included in our primary analysis. To test whether results were affected by differences between our scores from all nine items and those in the original LOT (all questions except ‘I'm a believer in the idea that "every cloud has a silver lining"’) or the LOT-R (questions marked (R) in the list above) we also calculated scores for the original LOT and LOT-R.

Optimism and pessimism scores were first computed separately from the sum of item scores, then combined:

$$ \begin{array}{ll} {{\text{Optimism}}\,{\text{score}}\, = \, \left( {{\text{Sum}}\,{\text{of}}\,{\text{scores}}\,{\text{for}}\,{\text{optimistic}}\,{\text{statements}}} \right)} \hfill & {{\text{Range}}\,{5}\,{\text{to}}\,{15}} \hfill \\ {{\text{Pessimism}}\,{\text{score}}\, = \,\left( {{\text{Sum}}\,{\text{of}}\,{\text{scores}}\,{\text{for}}\,{\text{pessimistic}}\,{\text{statements}}} \right) } \hfill & {{\text{Range}}\,{4}\,{\text{to}}\,{12}} \hfill \\ {{\text{Combined}}\,{\text{score}}\, = \, {\text{Optimism}}\,{\text{score}}\, + \,\left( {{16}{-}{\text{Pessimism}}\,{\text{score}}} \right)} \hfill & {{\text{Range}}\,{9}\,{\text{to}}\,{27}} \hfill \\ \end{array} $$

### Depression score

Depression was assessed using seven items from the Delusions-Symptoms-States-Inventory (DSSI)^20^. Each item is scored on a four-point scale of Not at all/A little/A lot/Unbearably. These responses were recoded to 0/1/2/3 and then summed across items for an overall depression score.

### National Death Index search

Names and dates of birth for study participants were submitted to the Australian National Death Index (NDI; see https://www.aihw.gov.au/about-our-data/our-data-collections/national-death-index/about-national-death-index) for matching against their records, as described in our previous publications^21,22^. The NDI records contain information about deaths in Australia from 1980 so deaths occurring outside Australia would not be matched through an NDI search. In some cases (for example, with common names or where date of birth is inaccurately estimated by relatives of the decedent) acceptable matches may not be achieved for people who have died. Identifying information was matched against deaths occurring in Australian States and Territories up to the end of October 2017, for a median period from completion of the questionnaire of 23.4 years. Matching occurred using an algorithm based on date of birth, and family and personal names weighted for frequency of names within the index (i.e. a match for an uncommon name was given greater weight than a match for a common name). On receipt of the search results, they were ranked according to matching score and re-checked for acceptability by a person experienced with NDI data.

For most deaths occurring before the end of 2016, an underlying cause of death and up to 12 other conditions present were reported. The reason for missing causes after that time is that information on date of death is received or coded earlier than the causes. Causes of death were coded by the NDI using the International Classification of Diseases, either ICD9 (up to 1996) or ICD10 (1997onwards). Only the ‘underlying cause of death’ was used in the cause-specific analysis. Causes were divided for the current analysis into three broad categories; cancers (malignant neoplasms: ICD9 codes 140-208, ICD10 codes C00-C97); cardiovascular diseases (diseases of the circulatory system: ICD9 codes 390-459, ICD10 codes I00-I99); and other known causes. Where no cause of death was available, the date of death was used in analysis of all-cause mortality and the case was censored at the date of death for the cause-specific analyses.

### Statistical methods

Survival analysis was based on scores and the date of death or censoring (recoded to age at death or at 21st October 2017). In the analysis of cause-of-death groups, censoring of participants who had died from causes other than those being examined was used rather than a subdistribution hazard model because our aim was to test for associations between mortality and scores, not to derive prognostic information on individuals’ survival^23^.

IBM SPSS, release 22 (IBM Corp., Amrok, NY) was used for data management, estimation of means and correlations and for preliminary survival analysis. However, because our recruitment of study participants emphasised twin-pairs, there is genetic overlap between many of the subjects. This means that, to the extent that participants are similar to each other for genetic reasons (which will vary according to the heritability of the characteristic under consideration), the effective number of independent observations is less than the number of participants and the standard errors for calculated statistics will be under-estimated. To overcome this problem, associations between scores and all-cause, any-cancer, any-cardiovascular, and other-cause mortality were tested using Cox regression in STATA (StataCorp LLC, College Station TX) with clustering by family to generate robust standard errors for the regression coefficients and confidence intervals for Hazard Ratios.

Consistency with the proportional hazards assumption of Cox regression was assessed using the STATA procedure ‘estat phtest’ and by plotting Schoenfeld residuals against the time variable (age at death or censoring). There was no evidence for deviation from proportional hazards for optimism, pessimism or depression scores (all *p* > 0.1, with no apparent slope in the residual plots).

To assess whether our conclusions would be similar if time since baseline was used as the time dimension, instead of age at death or censoring, we repeated the all-cause mortality analysis for pessimism score using time since baseline. Because the age of participants at baseline varied substantially, and this would affect survival, it was included as an additional covariate.

Estimation of the effects of genetic and environmental sources of variation on the optimism and pessimism scores, and on the correlation between them, was done using OpenMx (https://openmx.ssri.psu.edu/).

## Supplementary information

Supplementary information.

## Data Availability

Because of confidentiality assurances given to participants, raw data will not be available. Researchers interested in making further use of the data should enquire about possibilities for doing so during an approved visit to QIMR Berghofer Medical Research Institute.
