# Supplementary material for: Pessimism is associated with greater all-cause and cardiovascular mortality, but optimism is not protective
Source: Sci Rep. 2020 Jul 28;10:12609. doi: 10.1038/s41598-020-69388-y (PMC7387533; doi:10.1038/s41598-020-69388-y)

Pessimism is associated with greater all-cause and cardiovascular mortality,

but optimism is not protective.

John B Whitfield, Gu Zhu, J George Landers and Nicholas G Martin.

**SUPPLEMENTARY MATERIAL**

**Supplementary Table 1**. Heritability and genetic correlations for age-and sex-adjusted optimism and pessimism scores. Due to the skewed distributions of the scores (see Supplementary Figure 1 below), scores were either ranked or divided into three groups for this analysis. The Table shows proportions of variance attributable to additive genetic (A), dominance genetic (D) or shared-environmental (C), and non-shared environmental (E) sources; and phenotypic, additive genetic and non-shared environmental correlations between optimism and pessimism scores. Dropping the C component from the ACE model made no significant difference to the goodness-of-fit between the model and the data for either optimism or pessimism, but dropping D from the ADE model led to a significant deterioration for optimism. However both the AE and ADE models imply that around one-third of variance is genetic for both optimism and pessimism.

Note that the phenotypic correlations are derived from the maximum likelihood estimates and differ slightly from the rank correlation quoted in the text.

| OPTIMISM |  |  |  |  |  |  |  |  |  |  |  |  |  |  |
| --- | --- | --- | --- | --- | --- | --- | --- | --- | --- | --- | --- | --- | --- | --- |
|  |  |  |  |  |  |  |  |  |  |  |  |  |  |  |
| Variable | Model | Proportion of variance | | |  | Model fit | | |  | Model simplification | | | | |
|  |  | A | C \| D | E |  | -2LL | df | AIC |  | Starting  model |  | To | χ^2^ | p |
| Optimism (ranked scores) | ACE | 30.6 (21.4-37.5) | 0 (0-5.9) | 69.4 (62.5-76.8) |  | 7935.4 | 2815 | 2305.4 |  |  |  |  |  |  |
|  | AE | 30.6 (23.2-37.5) | - | 69.4 (62.5-76.8) |  | 7935.4 | 2816 | 2303.4 |  | ACE | Drop C | AE | 0 | 1.00 |
|  | CE | - | 19.5 (13.6-25.2) | 80.5 (74.8-86.4) |  | 7954.7 | 2816 | 2322.7 |  | ACE | Drop A | CE | 19.3 | 1.12 x 10^-5^ |
|  | ADE | 0 (0-23.1) | 34.2 (9.2-41.2) | 65.8 (58.8-73.4) |  | 7929.2 | 2815 | 2299.2 |  | ADE | Drop D | AE | 6.2 | 0.013 |
| Optimism  (three groups) | ACE | 32.4 (22.7-39.1) | 0 (0-6.8) | 67.6 (60.9-74.6) |  | 6784.5 | 2815 | 1154.5 |  |  |  |  |  |  |
|  | AE | 32.5 (25.4-39.1) | - | 67.5 (60.9-74.6) |  | 6784.5 | 2816 | 1152.5 |  | ACE | Drop C | AE | 0 | 1.00 |
|  | CE | - | 21.9 (16.1-27.5) | 78.1 (72.5-83.9) |  | 6803.9 | 2816 | 1171.9 |  | ACE | Drop A | CE | 19.4 | 1.06 x 10^-5^ |
|  | ADE | 0 (0-30.1) | 35.5 (3.4-42.1) | 64.5 (57.9-71.8) |  | 6779.8 | 2815 | 1149.8 |  | ADE | Drop D | AE | 4.7 | 0.030 |

| PESSIMISM |  |  |  |  |  |  |  |  |  |  |  |  |  |  |
| --- | --- | --- | --- | --- | --- | --- | --- | --- | --- | --- | --- | --- | --- | --- |
|  |  |  |  |  |  |  |  |  |  |  |  |  |  |  |
| Variable | Model | Proportion of variance | | |  | Model fit | | |  | Model simplification | | | | |
|  |  | A | C \| D | E |  | -2LL | df | AIC |  | Starting  model |  | To | χ^2^ | p |
| Pessimism (ranked scores) | ACE | 31.2 (17.2-37.8) | 0 (0-11) | 68.8 (62.2-75.8) |  | 7707.5 | 2738 | 2231.5 |  |  |  |  |  |  |
|  | AE | 31.2 (24.3-37.8) | 0 (0-0) | 68.8 (62.2-75.8) |  | 7707.5 | 2739 | **2229.5** |  | ACE | Drop C | AE | 0 | 1.00 |
|  | CE | 0 (0-0) | 22.9 (17.1-28.5) | 77.1 (71.5-83) |  | 7720.3 | 2739 | 2242.3 |  | ACE | Drop A | CE | 12.8 | 3.47 x 10^-4^ |
|  | ADE | 12.3 (0-36.8) | 20.4 (0-39.5) | 67.3 (60.4-74.7) |  | 7706.4 | 2738 | 2230.4 |  | ADE | Drop D | AE | 1.1 | 0.294 |
| Pessimism  (three groups) | ACE | 36.2 (17.6-42.5) | 0 (0-15.2) | 63.8 (57.5-70.6) |  | 6574 | 2738 | 1098 |  |  |  |  |  |  |
|  | AE | 36.2 (29.6-42.5) | 0 (0-0) | 63.8 (57.5-70.4) |  | 6574 | 2739 | **1096** |  | ACE | Drop C | AE | 0 | 1.00 |
|  | CE | 0 (0-0) | 27.4 (21.8-32.8) | 72.6 (67.2-78.2) |  | 6586.8 | 2739 | 1108.8 |  | ACE | Drop A | CE | 12.8 | 3.47 x 10^-4^ |
|  | ADE | 31 (0-42.4) | 5.6 (0-40.7) | 63.4 (56.8-70.4) |  | 6573.9 | 2738 | 1097.9 |  | ADE | Drop D | AE | 0.1 | 0.752 |

|  | Optimism-Pessimism Correlations | | |
| --- | --- | --- | --- |
|  | Phenotypic | Genetic | Environmental |
| Rank | -0.158 | -0.154 | -0.159 |
| 3 groups | -0.196 | -0.317 | -0.133 |

**Supplementary Table 2.** Sensitivity analyses; comparison of results for association between scores and all-cause mortality using differing approaches.

1. Comparison of the associations between pessimism, optimism and combined scores and all-cause mortality, based on all items (our primary analysis, expanded LOT) and scores based on the original LOT and revised LOT-R scales.

| Variable | HR | 95% CI | p-value |
| --- | --- | --- | --- |
| Pessimism score, LOT (4 questions) | 1.134 | 1.065 to 1.207 | 8.85E-05 |
| Pessimism score, LOT-R (3 questions) | 1.115 | 1.048 to 1.186 | 5.40E-04 |
| Optimism score, expanded LOT (5 questions) | 0.975 | 0.913 to 1.042 | 0.458 |
| Optimism score, LOT (4 questions) | 0.991 | 0.927 to 1.059 | 0.793 |
| Optimism score, LOT-R (3 questions) | 0.950 | 0.891 to 1.012 | 0.112 |
| Combined score, expanded LOT (9 questions) | 0.912 | 0.855 to 0.973 | 0.0056 |
| Combined score, LOT (8 questions) | 0.914 | 0.857 to 0.975 | 0.0063 |
| Combined score, LOT-R (6 questions) | 0.901 | 0.846 to 0.959 | 0.0011 |

In each case pessimism score is significantly associated with all-cause mortality and optimism score is not. The combined scores are each significantly associated with outcome, though with less significant p-values than the pessimism scores.

1. Comparison of results using age at death or censoring, or time from baseline (questionnaire completion) to death or censoring, as the time variable in Cox regression for all-cause mortality.

analysis time: age at event

|  | HR | 95% CI | p-value |
| --- | --- | --- | --- |
| Sex (Female = 0, Male = 1) | 1.478 | 1.289 to 1.696 | 2.27E-08 |
| Standardised residual of LOT (4-item) pessimism score | 1.134 | 1.065 to 1.207 | 6.26E-04 |

analysis time: length of followup

|  | HR | 95% CI | p-value |
| --- | --- | --- | --- |
| Sex (Female = 0, Male = 1) | 1.488 | 1.289 to 1.718 | 5.96E-08 |
| Age at baseline (per year) | 1.127 | 1.116 to 1.137 | (0) |
| Standardised residual of LOT (4-item) pessimism score | 1.117 | 1.044 to 1.195 | 0.0014 |

Using time from baseline to death or censoring as the time variable, and including age at baseline as a covariate, also shows a significant association between pessimism score and all-cause mortality but the HR is slightly closer to 1.00 and the p-value is less significant than when age is the time variable.

**Supplementary Figure 1**. Distributions of age- and sex-adjusted standardised residuals of pessimism and optimism scores.


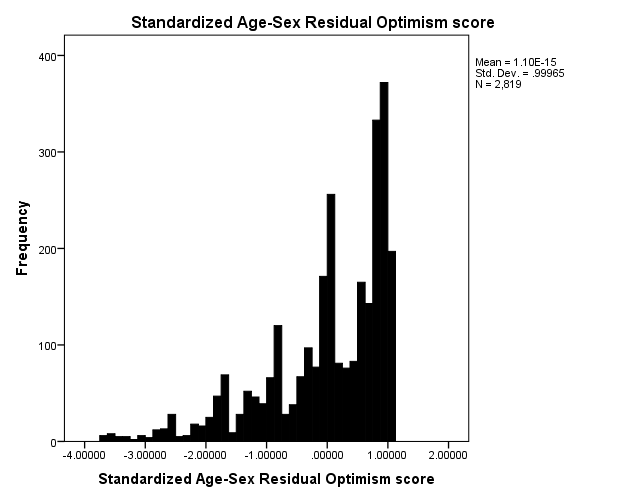


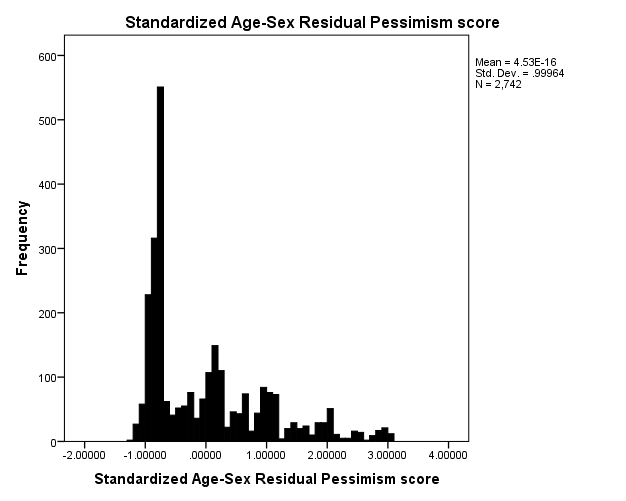

Supplement: Supplementary file 1 — Supplementary information. [file 41598_2020_69388_MOESM1_ESM.docx]
